# Examining American Adult’s Mental Well and Ill-Being During the 2020 COVID-19 Pandemic Using a Self-Determination Theory Perspective

**DOI:** 10.3389/ijph.2022.1604508

**Published:** 2022-04-14

**Authors:** Lauren N. Jordan, C. Veronica Smith

**Affiliations:** Department of Psychology, University of Mississippi, Oxford, MS, United States

**Keywords:** COVID-19, pandemic, wellbeing, self determination theory, need satisfaction, ill-being, need frustration, basic psychological needs

## Abstract

**Objectives:** The COVID-19 pandemic is an international health crisis that has caused unprecedented shifts in people’s environments and has threatened people’s wellbeing. The current study uses self-determination theory (SDT: 10) to understand how people were handling the pandemic, which proposes three basic psychological needs (autonomy, competence, and relatedness) are vital for human growth and thriving. Furthermore, we examined how people’s wellbeing and ill-being changed over the course of the pandemic.

**Methods:** A sample of 193 American adults from around the country reported on their need satisfaction and frustration as well as well and ill-being at three time periods during the pandemic (April 2020, late July/early August 2020, and late January/early February 2021).

**Results:** There was much variability in how people were handling the pandemic, but on average, wellbeing increased, and ill-being decreased over time. Consistent with SDT, the basic needs significantly predicted well and ill-being even during such unprecedented times of disruption.

**Conclusion:** Our results suggest that public health officials should help individuals to focus on autonomy, competence, and relatedness behaviors during times of upheaval.

## Introduction

The COVID-19 pandemic is an international health crisis which has infected over 33 million people and caused about 686,000 deaths in the United States alone as of September 2021 [1]. Prevention and mitigation efforts have led to an unprecedented shift in people’s environment, characterized by uncertainty and change in multiple domains [2]. People are making challenging choices, frequently with no obvious right or wrong answers, such as prioritizing oneself and health by staying at home or focusing on economics by supporting local restaurants and businesses. They are facing new ways of working, learning, and living, including having to adopt new procedures for their workplaces and daily living. They have tried to balance their need for belonging and social connection with practicalities, like social distancing requirements and travel restrictions.

As such, many individuals are experiencing incredible amounts of stress, anxiety, and depression [3]. Individuals’ negative functioning from before lockdowns (January to early March 2020) to after lockdowns (up to May 2020) in the United States, Europe, and Asia [4] and in Latin America [5] found that people’s anxiety and depression significantly increased [4]. Despite this, many people reported doing things to increase their wellbeing during the pandemic including spending more time outdoors [6], developing skills and hobbies [7], and hosting social gatherings over video calls [8]. Indeed, one study found that those participants who were told to focus on making meaningful choices, doing a task that was challenging but that they thought they could succeed at, and helping others during the pandemic, had higher subjective vitality and lower perceived stress compared to those participants who were not instructed to focus on these behaviors [9].

### Self-Determination Theory

One framework that explains variation in both ill-being (such as stress and depression) and wellbeing is the Basic Psychological Needs Theory of Self-determination Theory (SDT [10]). People have three basic psychological needs that must be satisfied to experience growth and wellbeing. Autonomy is the perception that any behavior in which an individual engages in is a result of their choice to behave that way, rather than a result of external factors and feelings of obligation. Competence is the perception of feeling effective in a certain context. Finally, relatedness is the perception of being listened to and cared for by others.

Satisfaction of these three basic psychological needs positively predicts hedonic wellbeing, the state of feeling good and being satisfied with life [11], and eudaimonic wellbeing, which involves personal growth, meaning, and purpose [12]. This relationship has been demonstrated with people of various ages, gender [12], and cultures [13]. It has also been demonstrated across numerous contexts, including the COVID-19 pandemic; individuals who reported that their autonomy, competence, and relatedness satisfaction increased during lockdowns experienced greater wellbeing compared to those who did not experience increases in their need satisfaction [14].

Individuals can also experience need frustration, which occurs when individuals feel prevented from feeling satisfied in their basic psychological needs. Researchers have found that need satisfaction in general positively predicts wellbeing (but need frustration was unrelated), and that need frustration positively predicts ill-being, such as depressive symptomology (but need satisfaction was unrelated) [13, 15]. One of the only papers that have investigated the unique influence of autonomy, competence, and relatedness satisfaction and frustration of well and ill-being within the same time point was conducted during COVID-19 pandemic (early April 2020) [16]. Although their results were mostly in line with prior research with need satisfaction uniquely predicting wellbeing and need frustration uniquely predicting ill-being, there were some exceptions. Both positive and negative affect predicted life satisfaction through competence frustration, but competence satisfaction was unrelated to life satisfaction. Similarly, relatedness frustration was not a consistent predictor of general distress. Importantly, this paper only examined one type of wellbeing and one type of ill-being, so questions remain as to the relationship with need satisfaction and frustration on well and ill-being and whether these relationships look different for multiple types of wellbeing and ill-being.

Investigating the three needs separately seems especially important during the COVID-19 pandemic. It could be that people’s autonomy feels especially threatened due to lockdown measures that prevent people from carrying out their normal routines, or that competence is especially threatened due to the amount of new information people are taking in regarding how to keep themselves safe, or that social distancing measures have people feeling particularly lonely and not related. Past research in self-determination theory suggests that people benefit from being fulfilled in all three needs regardless of the importance they place on that need, such as feeling a stronger desire to master skills (to feel competent) over relating to others [13]. Perhaps the pandemic has created a context in which satisfaction of one particular need (or frustration of a particular need) explains more or less variation in wellbeing (or ill-being). For example, Skewes et al. [17] studied faculty members undergoing the promotion and tenure process, which they described as an inherently low autonomy situation. However, many faculty members were still doing well if they were able to compensate for the low autonomy by focusing on ways to increase their competence and relatedness.

### Present Research

Although global pandemics are not new, multiple aspects of the COVID-19 pandemic make it unprecedented (e.g., transmissibility, economic impact) [18] and a worthy context in which to examine how established psychological theories perform in the prediction of mental health in extraordinary times. The aim of the current study was to examine the extent to which need satisfaction and frustration predicted mental health, consistent with SDT. We investigated several markers of both positive and negative functioning—hedonic wellbeing, or satisfaction with life, eudaimonic wellbeing, or growth and purpose, stress, and depressive thinking. Further, this study examined mental health over time, to support the existing literature that has focused on the beginning of the pandemic (e.g., 4).

## Methods

### Participants and Procedure

All study procedures were approved by the University’s Institutional Review Board and the study was pre-registered prior to data analysis.

Specifically, the measures and hypotheses/research questions were pre-registered after data was collected at Time 2 but before data was collected at Time 3 (https://aspredicted.org/w9ps5.pdf). Pre-registration was not done prior to Time 1 data collection because these data were originally collected as part of a larger dataset.

In April 2020, a sample of Americans adults was recruited through Prolific (a participant recruitment company) to complete an online survey, entitled “Understanding Daily American Life during a Pandemic” (Time 1). Participants were contacted again in late July/early August 2020 *via* Prolific and asked to participate in an originally unplanned follow-up study (Time 2) and one more time in late January/early February 2021 (Time 3). Participants were compensated with US$4.50 at Time 1, US$3.50 at Time 2, and US$2.50 at Time 3 (corresponding to an approximate hourly compensation rate of US$10.00 for each time point).

According to the U.S. Coronavirus Tracker [1], during the 3 days of Time 1 data collection, the average number of deaths per day was 1,746 with an average number of 31,036 new cases. During the 9 days of Time 2 data collection, the average number of deaths was 1,101 per day with an average number of 58,020 new cases. During the 11 days of Time 3 data collection, the average number of deaths was 3,165 with an average number of 129,193 new cases per day. The number of days the study was available in Time 1 was based on the amount of time it took us to reach our target participant rate of 400. This amount of time was higher in Times 2 and 3 to allow for more participants to participate.

The final sample represented a total of 193 participants who completed the study at all three time points and passed three or more of the four attention checks, representing a retention rate of 47% from the original 407 participants from Time 1. The sample size was chosen to match the availability of funds, but an a priori power analysis suggests that we would need about 150 participants to detect a medium effect size, indicating that we have adequate power to test our primary hypotheses. Demographics of this final sample can be found in Table 1. Participants in our sample were relatively diverse in terms of age, race, employment status, and geographic region.

**TABLE 1:** Demographic information for the sample (Examining American Adult’s Mental Well and Ill-Being During the 2020 COVID-19 Pandemic Using a Self-Determination Theory Perspective, United States, 2020).

Variable	N (%)	Mean (min/Max)
Age		52.16 (19/78)
Gender identity		
Male	86 (45)	
Female	103 (53.9)	
Transgender male	1 (0.5)	
Transgender female	0 (0)	
Gender variant/Non-conforming	0 (0)	
Prefer not to answer	1 (0.5)	
Racial identity		
White	138 (71.5)	
Black/African American	24 (12.4)	
Hispanic/Latino	4 (2.1)	
Native American/Alaskan Native	1 (0.5)	
Asian	11 (5.7)	
Native Hawaiian/Pacific Islander	0 (0)	
Multiracial	14 (7.3)	
Prefer not to answer	1 (0.5)	
Employment status at Time 3		
I am employed and working on location at my primary job	39 (20.2)	
I am employed and working remotely for my primary job	59 (30.6)	
I am employed but not currently working (e.g., furloughed)	3 (1.6)	
I am unemployed and lost my job due to the pandemic	15 (7.8)	
I am unemployed and was unemployed before the pandemic	26 (13.5)	
I am retired	49 (25.4)	
I do not work because I am a full-time student	2 [1]	
Geographic region		
Northeast (e.g., Massachusetts, New York, Vermont)	32 (16.7)	
Midwest (e.g., Michigan, Kansas, North Dakota)	40 (20.8)	
South (e.g., Mississippi, Texas, Virginia)	73 (38.0)	
West (e.g., Colorado, California, Alaska)	47 (25.5)	
COVID-19 vaccine status at Time 3		
Received COVID-19 Vaccine	7 (3.6)	
Made appointment for vaccine but not yet received	17 (8.9)	

*Note*. Geographic regions are based on US Census designations—https:// www.census.gov/programs-surveys/economic-census/guidance-geographies/levels.html.

In order to determine if participants who responded to all three time points differed from those we lost due to attrition, we conducted a series of independent sample *t*-tests. Our results indicated that at Time 1, all participants were relatively equally satisfied in their need satisfaction and in their hedonic wellbeing (all *t*-values < 1.42). However, those who failed to return after Time 1 had lower multi-faceted (combined hedonic/eudaimonic) wellbeing, greater ill-being, and had greater levels of COVID-19 experience, compared to those who responded to all three time points. Furthermore, participants who failed to return after Time 1 tended to be younger (all *t*-values > 2.12). Those who failed to return after Time 2 were equivalent to those who responded to all time points in need satisfaction and wellbeing, but had higher rates of autonomy and competence frustration, as well as stress (*t*-values > 2.00).

### Measures

Except for demographic variables (age, racial identity, gender, geographic region, employment status), all measures were assessed at all time points with higher scores indicating higher levels of the measured variable. Except for questions pertaining to experiences with COVID-19, all measures asked participants to reflect on the previous 7 days Reliability coefficients for all relevant measures can be found in Table 2.

**TABLE 2:** Descriptive statistics for all variables at all time points (Examining American Adult’s Mental Well and Ill-Being During the 2020 COVID-19 Pandemic Using a Self-Determination Theory Perspective, United States, 2020).

Variable	Cronbach’s α	M	SD	Range	Difference from midpoint of scale	
					t	*p*
Autonomy satisfaction						
Time 1	0.86	4.82	1.46	1–7	7.72	<0.001
Time 2	0.88	4.73	1.53	1–7	6.57	<0.001
Time 3	0.92	4.94	1.59	1–7	8.19	<0.001
Competence satisfaction						
Time 1	0.65	4.34	1.41	1–7	3.30	0.001
Time 2	0.67	4.32	1.45	1–7	3.05	0.003
Time 3	0.70	4.50	1.50	1–7	4.59	<0.001
Relatedness satisfaction						
Time 1	0.77	4.41	1.71	1–7	3.29	0.001
Time 2	0.87	4.27	1.78	1–7	2.13	0.035
Time 3	0.88	4.32	1.81	1–7	2.45	0.015
Autonomy frustration						
Time 1	0.67	3.37	1.38	1–7	−6.30	<0.001
Time 2	0.75	3.31	1.46	1–7	−6.55	<0.001
Time 3	0.70	3.21	1.43	1–7	−7.74	<0.001
Competence frustration						
Time 1	0.92	2.68	1.69	1–7	−10.90	<0.001
Time 2	0.92	2.81	1.67	1–7	−9.91	<0.001
Time 3	0.92	2.72	1.63	1–7	−10.92	<0.001
Relatedness frustration						
Time 1	0.68	3.01	1.63	1–7	−8.44	<0.001
Time 2	0.80	2.97	1.64	1–7	−8.77	<0.001
Time 3	0.80	3.03	1.73	1–7	−7.76	<0.001
Hedonic wellbeing						
Time 1	—	3.44	1.51	1–6	−0.60	0.552
Time 2	—	3.63	1.55	1–6	1.17	0.245
Time 3	—	3.67	1.56	1–6	1.52	0.129
Multi-faceted wellbeing						
Time 1	0.88	3.46	1.14	1.2–6	−0.48	0.631
Time 2	0.89	3.60	1.16	1–6	1.20	0.230
Time 3	0.90	3.62	1.18	1.2–6	1.42	0.158
Perceived stress						
Time 1	0.77	2.59	0.92	1–5	−6.12	0.001
Time 2	0.78	2.50	0.94	1–5	−7.49	0.001
Time 3	0.78	2.45	0.94	1–5	−8.15	0.001
Depressive outlook						
Time 1	0.88	3.27	1.34	1–6.67	−7.56	<0.001
Time 2	0.91	3.23	1.43	1–7	−7.47	<0.001
Time 3	0.92	3.14	1.46	1–7	−8.16	<0.001
COVID-19 experience						
Time 1	—	0.39	0.82	0–3	−35.26	<0.001
Time 2	—	0.87	1.12	0–4	−20.01	<0.001
Time 3	—	1.62	1.34	0–5	−9.01	<0.001

*Note.* Cronbach’s α was not calculated for hedonic wellbeing because it is a single item measure. Cronbach’s α was not calculated for COVID-19 experience because it is a count variable.

#### Need Satisfaction

Participants responded to six items originally created by Sheldon et al. [20], with two items each referencing satisfaction of autonomy (e.g., “My choices were based on my own interests and values”), competence (“Very capable in what I did”), and relatedness (“Close and connected with other people”) with a 7-point scale (1 = *not at all true*; 7 = *very true*). Only two items were chosen in order to reduce participant demand.

#### Need Frustration

Using a 7-point scale (1 = not at all true; 7 = very true), participants responded to eight statements written for the current study. These items were adapted from the need satisfaction measure by Sheldon et al. [20], and were designed to be relevant to most people during the COVID-19 pandemic: autonomy frustration (3 items: “I felt limited in the choices I was able to make”), competence frustration (3 items: “I questioned by abilities”), and relatedness frustration (2 items: “I did not feel close to the people I care about”).

#### Wellbeing Measures

##### Hedonic Wellbeing

Because satisfaction is a crucial element of hedonic wellbeing [21] we used a single-item measure of life satisfaction. Participants responded using a 6-point scale (1 = at no time; 6 = all of the time) to a single item, “Did you feel satisfied with your life?” Single-item measures of life satisfaction have been shown to be strongly correlated with multiple-item measures and to have high test-retest reliability [22].

##### Multi-Faceted Wellbeing

Participants responded to the five-item World Health Organization Wellbeing Index [23] using a 6-point scale (1 = at no time; 6 = all of the time). These items assess both hedonic wellbeing (e.g., “Did you feel cheerful and in good spirits,” as well as eudaimonic wellbeing (e.g., “Did you feel as if your daily life was filled with things that interested you?”).

#### Ill-Being Measures

##### Depressive Outlook

Participants responded to three items from Gable and Nezlek [24] representing Beck’s cognitive triad [25], or negative views about self, current world, and future: 1) “Overall, how positively did you feel about yourself over the past 7 days?” 2) “Thinking of your life in general, how well did things go over the past 7 days?” and 3) “How optimistic are you about how your life (in general) will be in the next 7 days?” using 3 unique 7-point bipolar scales (e.g., for Item (c), 1 = very pessimistic; 7 = very optimistic). All three items were reverse scored to allow for higher scores to indicate higher depressive outlook.

##### Perceived Stress

Participants completed the Perceived Stress Scale—4 item (PSS-4; [26]) (e.g., “...that things were going your way” (reverse scored), “...that you were unable to control important things in your life”) using a 5-point scale (1 = not at all; 5 = extremely).

#### COVID-19 Experience

Participants were asked if they had been tested for COVID-19 (1 item), had been diagnosed with COVID-19 (1 item), or knew anyone who had COVID-19 (3 items). Participants responded to each item by indicating either “yes” (coded as 1), “no” (coded as 0) or “not applicable” (removed from this total). Responses for all 5 items were summed to create a composite score of COVID experience.

## Results

The means, standard deviations, and ranges for all variables at Times 1, 2, and 3 are in Table 2. Common method bias can occur if both predictor and outcome variables are collected via the same method such as self-report and is concerning as it can lead to biased parameter estimates. As such, we conducted the Harman's one factor test which assesses the degree of bias that may be present by conducting a factor analysis with all predictor and outcome items and assessing how much variance is explained by the first factor in the analysis. Fuller et al. ^20^ suggests that parameter estimates become biased at around 60% of variance being explained by the first factor. At each time point, our factor analysis suggested that the first factor did not explain more than 46% of variance at each time point which suggests that our common methods are likely not inflating our reported parameter estimates.

### Wellbeing and Ill-Being During the Pandemic

All variables (except COVID-19 experience at Time 1 and Time 2) spanned mostly the full range of possible values and were relatively normally distributed, indicating that participants varied widely in how they were coping with the pandemic at all three time points, with most participants scoring around the mean of the distribution.

A series of one-sample t-tests examined whether participants scored significantly above or below the mid-point of each measure of well and ill-being (Table 2). Need satisfaction scores were higher than the midpoint at all time points and ill-being and need frustration scores were significantly lower than the midpoint at all time points. Surprisingly, wellbeing scores were not different from the midpoint at any time point.

### Predicting Well and Ill-Being With Need Satisfaction and Frustration

#### Analytical Overview

We first report bivariate correlations between all variables and for all three time points (Table 3). We then report on a series of multilevel random coefficient models using HLM 8 [27], with the three periods of time during the pandemic nested within people. This analytic strategy allowed us to reduce our family-wise error by investigating all variables of interest (i.e., time, need satisfaction and frustration) in the same model [28]. First, a series of unconditional models on each of our well and ill-being outcome variables estimated the intraclass correlation (ICC), or the percentage of variability in each outcome variable due to within-person differences, or differences between time points. A significant amount of variance due to within-person/between time point differences also indicates that multi-level analysis is appropriate to disentangle within- and between-person effects. Second, we modeled differences in well/ill-being based on the period of time during the pandemic, satisfaction and frustration of autonomy, competence, and relatedness, and COVID-19 experience, all at Level 1. Level 2 was unconditional, with no added predictors (Table 4). Our Level 1-only model is shown below:

yij=β0j+β1j(Time)+β2j(Autonomy Satisfaction)+ β3j(Competence Satisfaction)+β4j(Relatedness Satisfaction)+β5j(Autonomy Frustration)+β6j(Competence Frustration)+β7j(Relatedness Frustration)+β8j(COVID−19 experience)+ rij

**TABLE 3:** Correlation matrix of time 1, 2, and 3 need satisfaction, need frustration, wellbeing, and ill-being (Examining American Adult’s Mental Well and Ill-Being During the 2020 COVID-19 Pandemic Using a Self-Determination Theory Perspective, United States, 2020).

	1	2	3	4	5	6	7	8	9	10
1. Autonomy satisfaction										
Time 1	—									
Time 2	—									
Time 3	—									
2. Competence satisfaction										
Time 1	0.53	—								
Time 2	0.63	—								
Time 3	0.64	—								
3. Relatedness satisfaction										
Time 1	0.39	0.47	—							
Time 2	0.52	0.55	—							
Time 3	0.49	0.53	—							
4. Autonomy frustration										
Time 1	−0.37	−0.23	−0.29	—						
Time 2	−0.46	−0.40	−0.38	—						
Time 3	−0.45	−0.41	−0.39	—						
5. Competence frustration										
Time 1	−0.34	−0.46	−0.40	0.68	—					
Time 2	−0.54	−0.58	−0.56	0.73	—					
Time 3	−0.52	−0.62	−0.48	0.73	—					
6. Relatedness frustration										
Time 1	−0.26	−0.33	−0.54	0.56	0.61	—				
Time 2	−0.50	−0.50	−0.74	0.54	0.71	—				
Time 3	−0.44	−0.48	−0.76	0.59	0.68	—				
7. Hedonic wellbeing										
Time 1	0.47	0.55	0.46	−0.40	−0.50	−0.43	—			
Time 2	0.58	0.60	0.59	−0.46	−0.61	−0.58	—			
Time 3	0.60	0.55	0.57	−0.59	−0.60	−0.55	—			
8. Multi-faceted wellbeing										
Time 1	0.52	0.60	0.43	−0.48	−0.60	−0.43	0.79	—		
Time 2	0.65	0.60	0.64	−0.55	−0.66	−0.63	0.78	—		
Time 3	0.61	0.64	0.57	−0.57	−0.62	−0.58	0.80	—		
9. Perceived stress										
Time 1	−0.41	−0.45	−0.44	0.62	0.70	0.54	−0.66	−0.67	—	
Time 2	−0.57	−0.54	−0.53	0.61	0.71	0.59	−0.72	−0.71	—	
Time 3	−0.56	−0.53	−0.53	0.68	0.75	0.59	−0.73	−0.72	—	
10. Depressive outlook										
Time 1	−0.45	−0.52	−0.40	0.57	0.68	0.56	−0.66	−0.71	0.82	—
Time 2	−0.62	−0.61	−0.59	0.57	0.72	0.64	−0.82	−0.78	0.83	—
Time 3	−0.64	−0.63	−0.60	0.62	0.76	0.62	−0.80	−0.80	0.83	—
11. COVID-19 experience										
Time 1	−0.06	−0.00	0.05	0.07	0.02	0.03	−0.08	−0.09	0.14	0.08
Time 2	0.01	0.09	0.16	0.11	0.04	−0.05	0.01	−0.04	−0.01	−0.05
Time 3	−0.03	0.01	0.10	0.04	0.07	−0.01	0.09	−0.02	0.04	−0.02

*Note*. Values greater than or equal to .16 are significant at *p* < 0.05. All values greater than 0.20 are significant at *p* < 0.01.

**TABLE 4:** Multi-level model results (Examining American Adult’s Mental Well and Ill-Being During the 2020 COVID-19 Pandemic Using a Self-Determination Theory Perspective, United States, 2020).

	Hedonic wellbeing	Multi-faceted wellbeing	Perceived stress	Depressive outlook
	*b* (SE)	*b* (SE)	*b* (SE)	*b* (SE)
Intercept	3.47 (0.14)*	3.42 (0.10)*	2.63 (0.08)*	3.25 (0.13)*
Time	0.11 (0.05)*	0.07 (0.03)*	−0.06 (0.03)*	−0.02 (0.05)
Autonomy satisfaction	0.12 (0.04)*	0.10 (0.03)*	−0.05 (0.02)*	−0.11 (0.04)*
Competence satisfaction	0.12 (0.05)*	0.09 (0.04)*	−0.06 (0.03)	−0.14 (0.05)*
Relatedness satisfaction	0.13 (0.05)*	0.11 (0.04)*	−0.03 (0.03)	−0.05 (0.05)
Autonomy frustration	−0.06 (0.04)	−0.11 (0.04)*	0.11 (0.04)*	0.15 (0.06)*
Competence frustration	−0.06 (0.05)	−0.09 (0.05)*	0.16 (0.04)*	0.14 (0.07)*
Relatedness frustration	−0.06 (0.04)	−0.05 (0.04)	0.05 (0.03)	0.14 (0.05)*
COVID-19 experience	0.05 (0.04)	−0.00 (0.03)	−0.02 (0.03)	−0.04 (0.04)

Note: *p ≤ 0.051. Coefficients with robust standard errors are reported.

Except for the time variable, all coefficients were group-mean centered, representing fluctuations relative to one’s own average score of that predictor variable. The intercept of each model represents the predicted wellbeing/ill-being score when all other variables are at the mean for each person and when Time = 0 (in other words, Time 1). The coefficient for time $\;\left[\beta_{1j}\left(Time\right)\right]$ , which was uncentered, represents the degree of changes in well/ill-being for each increase in time (while controlling for all other variables). All other coefficients can be interpreted as changes in well/ill-being for each one-point change from each person’s mean on that predictor.

#### Bivariate Correlations

A correlation matrix of all predictor and outcome variables is shown in Table 3 for all time points. All predictor variables were significantly correlated with one another in the expected direction, with need satisfaction positively predicting wellbeing and negatively predicting ill-being, and need frustration negatively predicting wellbeing, and positively predicting ill-being. Correlation sizes ranged from small/medium (*r* = 0.20) to large (*r* = −0.86).

#### Within-Person Variability in Wellbeing and Ill-Being

The ICCs for all outcome variables were statistically significant at *p* < 0.001, indicating that a significant portion of variance in each outcome variable was due to within-person or between-time point variance. Regarding wellbeing measures, the ICC was smallest for life satisfaction (ICC = 0.20) indicating that about 20% of variance in life satisfaction was due to people changing over time, while about 80% of variance was due to differences between individuals. About 30.8% of variance in multi-faceted wellbeing was due to people changing over time. The ICCs were slightly higher for both ill-being measures as 34.7% of variance in stress and 35.7% in depression were explained by time-point differences.

Given the significant ICC coefficients, we next tested for the relationship between need satisfaction, need frustration, and time with all outcome variables (Table 4). In all analyses, COVID-19 experience was entered as a covariate. Of note, it was not a significant predictor of any outcome variable.

#### Wellbeing

##### Hedonic Wellbeing

Time was a significant and positive predictor of hedonic wellbeing, indicating that at every subsequent time point beyond the first, hedonic wellbeing increased by about 0.11 points while controlling for need satisfaction, need frustration, and COVID-19 experience. Autonomy, competence, and relatedness satisfaction all positively and significantly predicted hedonic wellbeing and were similar in magnitude, indicating that for every 1-point increase above an individual’s score for that predictor averaged across time points, hedonic wellbeing increased by 0.12, 0.12, and 0.13 points, respectively. Need frustration was unrelated to hedonic wellbeing.

##### Multi-Faceted Wellbeing

Time was a significant positive predictor of multi-faceted wellbeing, as were autonomy, competence, and relatedness satisfaction. In addition, both autonomy and competence frustration were significantly associated with lower multi-faceted wellbeing. All statistically significant coefficients were roughly similar in magnitude, indicating that the strength of the relationship between satisfaction and frustration of each need with multi-faceted wellbeing was about the same.

#### Ill-Being

##### Perceived Stress

Time significantly and negatively predicted stress, which means that people’s perceived stress lessened over the course of the pandemic. Autonomy satisfaction negatively predicted stress. Autonomy and competence frustration were significantly associated with more perceived stress and these coefficients were slightly stronger in magnitude when compared with autonomy satisfaction.

##### Depressive Outlook

Unlike stress, time was not a significant predictor of depressive outlook. Autonomy and competence satisfaction predicted lower depressive outlook, while relatedness satisfaction was unrelated. All three need frustration variables predicted greater depressive outlook and all significant predictor variables were roughly similar in magnitude.

## Discussion

Our results indicate that in general, people were doing relatively well during all three time periods in the pandemic. People’s wellbeing generally improved over time while stress generally decreased over time. Depressive outlook remained relatively stable throughout the pandemic. Although people were, on average, doing relatively well during the pandemic, it is important to keep in mind that there was still a great deal of variance in people’s experiences—some individuals reported thriving, while others reported struggling, consistent with other current research regarding wellbeing and ill-being during the beginning of the pandemic [3].

Importantly, mental health (both well and ill-being) was predicted by need satisfaction and frustration, consistent with SDT; models that included these variables accounted for significant amounts of variance in all indices of well and ill-being. In line with SDT and our expectations, satisfaction of autonomy, competence, and relatedness were each unique predictors of both hedonic and multi-faceted wellbeing and predicted wellbeing at relatively equal rates. That is, people tended to be higher in wellbeing during those time periods in the pandemic in which they were also higher in satisfaction of each of their basic psychological needs; time periods in which they were less satisfied with their needs was associated with lower wellbeing. Our results were also in line with what we would expect for need frustration; time periods in which people felt frustrated in their needs of autonomy, competence, and relatedness were generally associated with higher ill-being, most notably for depressive outlook.

Critically, our findings also point to the importance of concurrent consideration of need frustration and satisfaction and the importance of examining multiple types of well and ill-being. Although others have found that satisfaction uniquely predicts wellbeing and frustration uniquely predicts ill-being [13], our results were not entirely consistent with these previous findings. Beyond the significant bivariate correlations between need frustration and all indices of well and ill-being, autonomy and competence frustration emerged as significant and unique predictors amongst both wellbeing and ill-being measures, even when controlling for need satisfaction and COVID-19 experience. The opposite was also true—need satisfaction variables were associated with stress and depressive outlook, not just wellbeing, even while controlling for need frustration. These results indicate that need frustration is not just the inverse of need satisfaction, and that both should be examined in order to better explain and predict differences in well and ill-being.

We were also interested in whether satisfaction or frustration of a particular need seemed to be especially important during the pandemic. Although all significant predictors were similar in magnitude within outcome variables, some seemed especially important when comparing across outcome variables. Autonomy satisfaction predicated all of the outcome variables. This is perhaps unsurprising as restrictions such as stay-at-home orders have changed much of the way that people are living, and those individuals who still found ways to make choices about the things that they enjoy were likely also doing especially well. Both competence satisfaction and competence frustration were also relatively consistent across outcome variables, which could be because much of the pandemic was characterized by uncertainty, change, and new ways of living and working. By contrast, relatedness satisfaction and frustration did not serve as unique predictors of well or ill-being, despite many concerns about the pandemic’s negative effects on relationships (e.g. [29]) and increases in loneliness and isolation (e.g., [30]).

### Future Directions and Limitations

The results of the current study raise several important questions that we encourage future research to consider. For example, it is unclear whether people were especially sensitive to changes in autonomy and competence during the COVID-19 pandemic, a time characterized by increased regulation and much uncertainty, or whether similar results would emerge outside of the pandemic. Research should continue to investigate whether there are various contexts that influence the extent to which individuals are especially frustrated in their autonomy, competence, or relatedness. Notably, factors directly concerning the pandemic influence differences in wellbeing, such as the degree of isolation that people experience during lockdowns and the number of days that individuals have been in lockdowns [14] and certainly factors not directly related to the pandemic influence wellbeing as well such as meaning in life [31] sleep quality [32], financial wellbeing [33], and even travel [34]. Because these were all impacted during the pandemic to varying degrees, future research should investigate the downstream effects of these factors on wellbeing as well as their unique effects on need satisfaction and frustration.

Although the current study adds to our understanding of both the COVID-19 pandemic and SDT, the findings should be considered in light of several limitations. Using an online research platform allowed us to gather a sample from around the country and such samples have generally been found to provide quality data (e.g., [35]); however, the types of people providing data online during a pandemic may differ in important ways from the general population (e.g., access to the internet, more time at home). Additionally, our attrition analyses indicated that the participants who failed to return after Time 1 were doing less well than those who responded to all three time points. This may partially account for the fact that participants in our sample generally reported higher than average levels of need satisfaction and lower than average need frustration. However, our data did span mostly the full range of values for every variable, which indicates that there was still high variance in our participants’ experiences.

Participants were also asked to reflect on the preceding week when completing measures, which may or may not have adequately captured the full variability in people’s lives. Research conducted during the pandemic has shown that people’s daily experiences do vary (even during lockdowns) and that these experiences have differential impacts on wellbeing [7]. Consistent with previous work that has examined daily variability in SDT needs and outcomes (e.g., [31, 36]), future research would benefit from the use of experience sampling techniques in order to capture events that predict both need satisfaction and frustration, as well and ill-being.

### Conclusion

The COVID-19 pandemic has led to dramatic shifts in much of life, including how individuals work, maintain relationships, and accomplish daily tasks. Our participants reported a wide range of mental health experiences during the pandemic, with most doing relatively well. Satisfaction and frustration of autonomy, competence, and relatedness explained significant amounts of variance in multiple indices of wellbeing and ill-being. Of particular interest was the unique importance of autonomy satisfaction, which predicted more wellbeing and less ill-being. This may indicate that it is important for people to feel a sense of choicefulness, especially during periods of high restriction which serve to protect the health and safety of everyone. Public health experts may try to encourage people to maintain choicefulness, finding things that they intrinsically enjoy doing, especially during lockdowns when autonomy may be especially threatened. Of course, it is important to recognize that maintaining choicefulness will be easier for some, such as those who have access to appropriate resources, and that both psychological needs and physical needs are predictive of mental health [37]. In sum, our findings represent an important addition in our understanding of mental health during the COVID-19 pandemic, as well as an important contribution to the self-determination theory literature regarding the relationship between need frustration and well and ill-being.
